# KATANIN is required for the formation of complete preprophase bands and subsequent division positioning in two asymmetric divisions of the maize stomatal complex

**DOI:** 10.1242/jcs.265055

**Published:** 2026-09-10

**Authors:** Stephanie E. Martinez, Carolyn G. Rasmussen

**Affiliations:** Department of Botany and Plant Sciences, Center for Plant Cell Biology, University of California, Riverside, CA 92521, USA

**Keywords:** Cell division, Microtubules, Cytoskeleton, Katanin, Maize, Preprophase band, Phragmoplast, Nucleus, Nuclear position

## Abstract

Microtubule dynamics underpin cell division, movement and growth in eukaryotic organisms. KATANIN p60 is a microtubule-severing protein that promotes proper cell elongation and division. In *Zea mays* (maize), p60 is encoded by two genes, *Discordia3a (Dcd3a)* and *Dcd3b*. Here we refer to maize *dcd3a dcd3b* double mutants as p60 mutants. In plants, division positioning is initiated in late G2 via the formation of a microtubule structure called the preprophase band (PPB). Maize p60 mutants have defects in microtubule severing and form abnormal PPBs in symmetrically dividing cells, including both incompletely assembled and misoriented PPBs. Here, we show that two types of asymmetric divisions required to generate stomatal complexes in maize p60 mutants have normally positioned but often incompletely formed PPBs. Incompletely formed PPBs lead to misoriented divisions and nuclear positioning defects in p60 mutants.

## Teaser

**Highlighted Article:** Division positioning defects in two asymmetric divisions required for stomatal complex formation in maize p60 mutants are due to incomplete, but not misoriented, preprophase band formation.

## INTRODUCTION

Microtubule dynamics and organization are important for many cellular processes, including division, elongation and differentiation (Hashimoto, 2015; Horio and Murata, 2014). Microtubule severing is essential for microtubule reorganization and is performed in many eukaryotes by ‘ATPases associated with diverse cellular activities’ (AAA-type) ATPases like KATANIN, a complex composed of p60 and p80 subunits. The p80 subunit recruits the p60 to microtubules for p60-mediated ATP-dependent microtubule severing (Deinum et al., 2017; Hartman and Vale, 1999; Hartman et al., 1998; McNally and Vale, 1993; Wang et al., 2017; Zhang et al., 2013). In animals, p60-mediated microtubule severing is important for completion of cytokinesis (Matsuo et al., 2013), meiosis (Dunleavy et al., 2023; Lynn et al., 2021; Srayko et al., 2000) and many other processes (Ghosh et al., 2012). In plants, p60 mutants have aberrant microtubule organization, leading to shorter, wider cells and small organs (Attrill and Dolan, 2024; Bichet et al., 2008; Burk et al., 2001; Komis et al., 2017; Komorisono et al., 2005; Luptovčiak et al., 2017; Martinez et al., 2026; Panteris and Adamakis, 2012; Wang et al., 2021). In *Zea mays* (maize), p60 is encoded by two genes, *Discordia3a* (*Dcd3a*) and *Dcd3b,* both of which play mostly redundant roles in growth and fertility (Martinez et al., 2026).

**Fig. 1.**
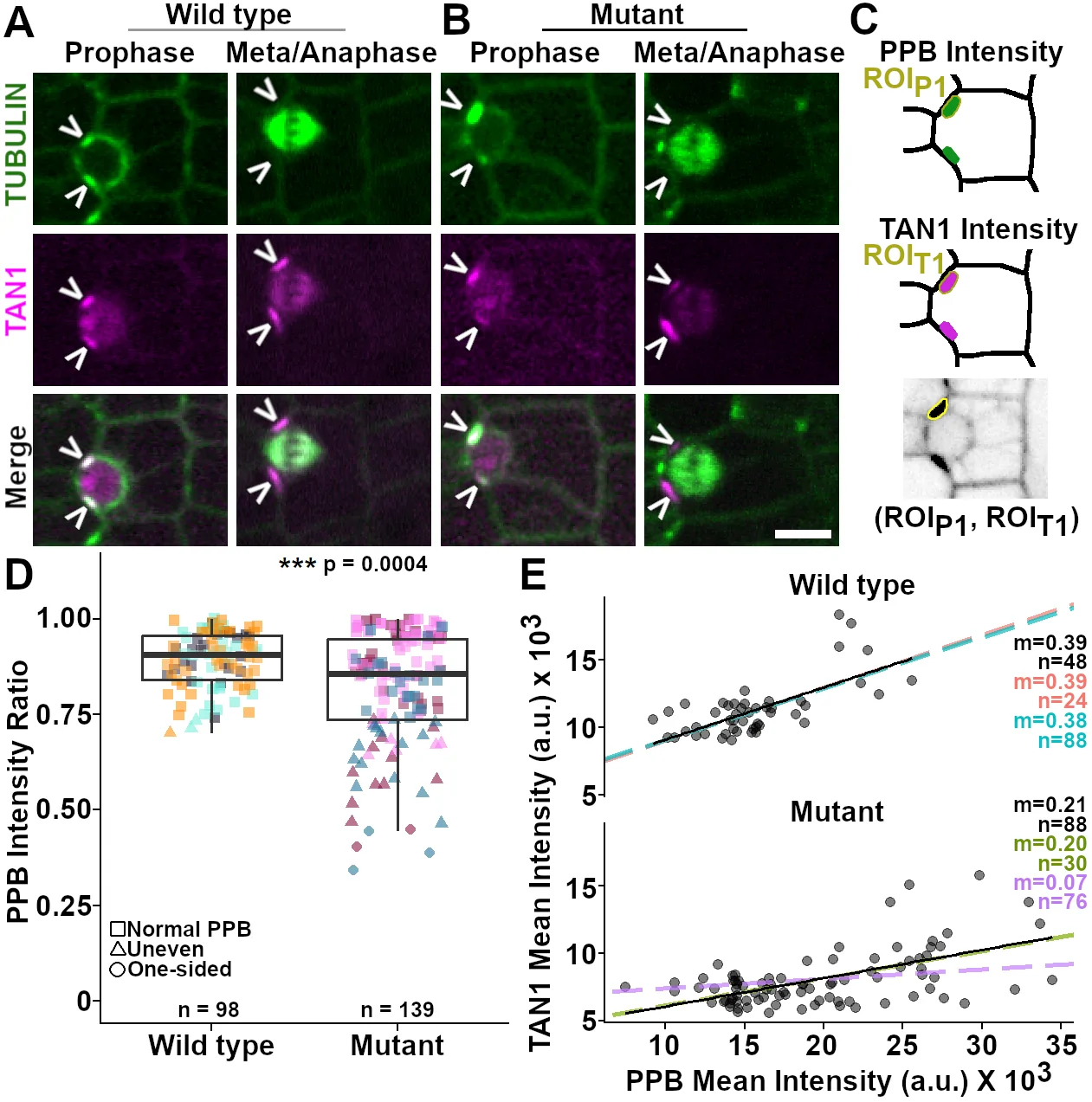
**TAN1 is a proxy for PPB accumulation.** (A,B) Representative micrographs of asymmetrically dividing subsidiary mother cells expressing CFP–TUBULIN (microtubules, green) and TAN1–YFP (magenta) for (A) wild-type cells (*dcd3a-2/+; dcd3b-1/+*) with equivalent accumulation of both microtubules and TAN1-YFP and (B) p60 mutant cells (*dcd3a-2; dcd3b-1*) with uneven accumulation of both TAN1–YFP and microtubules at each side of the division site. White arrowheads indicate the division site. All images are the same scale. Scale bar: 10 μm. (C) Schematic showing the division site where fluorescent intensities were measured. (D) PPB fluorescence intensity ratio from three wild-type and mutant plants, indicated with different color dots. Dot shape, PPB type. Cell counts are shown. *P*-value shown calculated from Wilcoxon rank sum test. Boxplot shows median, first and third quartiles. Whiskers represent values within 1.5× the interquartile range from the first and third quartiles boundaries. (E) Scatter plots comparing relative TAN1 and PPB mean intensity from cells in late G2/prophase. Three plants per genotype were analyzed. Raw data from one plant consists of black dots corresponding to individual fluorescence intensity measurements (with a black line of best fit); colored dotted lines show best fit of two other plants. m, slope; n, number of division site fluorescence intensities measured. a.u., arbitrary units.

In grasses, such as maize and *Brachypodium distachyon*, stomatal complexes have two guard cells flanked by two subsidiary cells (Berg and Raissig, 2025; Gray et al., 2020; Stebbins and Shah, 1960). Subsidiary cells are formed from an asymmetric division that generates a small subsidiary cell directly in contact with the guard cell mother cell and a pavement cell. The division positioning is very consistent in these cells (Guo et al., 2021; McKown and Bergmann, 2020). Before the onset of cell division, the nucleus polarizes adjacent to the guard mother cell for the asymmetric division of the subsidiary mother cell (Facette et al., 2019). Nuclear polarization is mediated by the proper localization of BRICK1, which recruits the leucine-rich-repeat receptor-like kinase PANGLOSS2 (PAN2; reviewed in Zhou et al., 2026). PAN2 recruits the WEAK CHLOROPLAST MOVEMENT UNDER BLUE LIGHT 1 (WEB1)/PLASTID MOVEMENT IMPAIRED 2 (PMI2)-RELATED (WPR) proteins, which interact directly with F-actin (Nan et al., 2023). PAN2 also recruits PANGLOSS1 (PAN1) and plant-specific Rho-of-plants (ROP) GTPases to enhance actin patch accumulation at the interface between the subsidiary mother cell and the guard cell (Facette et al., 2015; Humphries et al., 2011; Zhang et al., 2012). The maize LINC KASH SINE-LIKE2 (MLKS2) mediates the polarized movement and tethering of the nucleus to the actin patch (Ashraf et al., 2023). Then, during late G2, the preprophase band (PPB), a cortical ring of microtubules and actin filaments, forms and marks the division site, or the location of the division after the completion of cytokinesis (Fig. 1A).

The PPB recruits a suite of proteins, of which a small subset remains at the division site after PPB disassembly, including the microtubule-binding protein TANGLED1 (Bellinger et al., 2023; Livanos and Müller, 2019; Martinez et al., 2017; Rasmussen et al., 2011; Smith et al., 2001; Uyehara and Rasmussen, 2023; Uyehara et al., 2024; Walker et al., 2007). Live-cell timelapse imaging has demonstrated that TAN1 localization at the division site during later stages of mitosis indicates prior PPB accumulation. Therefore, TAN1 division site localization is an excellent proxy for earlier PPB accumulation (Uyehara et al., 2024).

In maize, *Marchantia polymorpha* and Arabidopsis, p60 mutants have a combination of incompletely formed PPBs, misoriented PPBs and division plane orientation defects (Attrill and Dolan, 2024; Komis et al., 2017; Martinez et al., 2026; Ovečka et al., 2020; Panteris et al., 2011; Webb et al., 2002). Following PPB disassembly, the mitotic spindle forms in metaphase and separates chromosomes in anaphase (Fig. 1A). In telophase, the phragmoplast, an anti-parallel microtubule array, expands towards the division site and facilitates the formation of the cell plate, which will mature into the cell wall that separates the two daughter cells (Müller and Jürgens, 2016; Rasmussen and Bellinger, 2018; Samuels et al., 1995). Phragmoplasts often grow with arrowhead-shaped aberrantly oblique leading edges in p60 mutants (Komis et al., 2017; Martinez et al., 2026; Panteris et al., 2011). Near the cell cortex, cortical telophase microtubules, which are stabilized by TAN1 or other division-site localized proteins, are added into the phragmoplast to direct phragmoplast positioning (Bellinger et al., 2023). In p60 mutants, delayed microtubule re-organization leads to defects in cell wall formation during cytokinesis (Panteris et al., 2021).

In symmetric divisions, maize p60 mutants (*dcd3a-2; dcd3b-1* double mutants) form both incomplete and misoriented PPBs. Incomplete PPBs, containing uneven or one-sided microtubule accumulations at the expected division site, do not lead to misoriented divisions. Only rarely occurring (5%) misoriented PPBs, containing microtubule accumulations not at the division site but in another aberrant location, lead to misoriented symmetric divisions in p60 mutants. Subsidiary cells are occasionally misoriented ∼15% (Martinez et al., 2026). Misoriented subsidiary mother cell PPBs have been observed in maize *brick1* mutants (Panteris et al., 2006), whereas *Brachypodium distachyon* (*Bd*)*polar* mutant subsidiary mother cells sometimes have additional division sites specified by *BdTAN1* (Zhang et al., 2022). We hypothesized that, similar to symmetric divisions in p60 mutants, misoriented asymmetric divisions would be caused by misoriented or ectopic PPBs. In contrast to our hypothesis, there were no misoriented PPBs in two asymmetric divisions, subsidiary mother cell divisions and guard mother cell precursor divisions. Instead, incomplete PPBs generated misoriented divisions.

## RESULTS AND DISCUSSION

To determine when defects arise during asymmetric division, we performed live-cell imaging of p60 mutants (*dcd3a-2; dcd3b-1* homozygous double mutants) and wild-type siblings (*dcd3a-2/+; dcd3b-1/+*) expressing CFP–TUBULIN and TAN1–YFP. First, we assessed PPB positioning in subsidiary mother cell divisions. There were neither misoriented PPBs nor cells completely lacking PPBs in either mutants or wild-type plants (*n*=3 plants per genotype, ≥98 cells analyzed). Next, we assessed whether PPBs were normal or incomplete. We defined normal PPBs as those with uniform fluorescence intensities on both sides of the division site at the midplane of the cell with PPB intensity ratios from 1 to 0.74 (Fig. 1A,C,D). Normal PPBs were observed 97% of the time in wild-type plants (*n*=95/98 cells from three plants) and 73% of the time in p60 mutants (*n*=102/139 cells from three plants). The rest of the PPBs in the p60 mutant were either uneven (∼23%, *n*=32/139 cells fluorescence intensity ratio between 0.45–0.74 at the division site, Fig. 1B) or one-sided (∼4%, *n*=5/139 cells with fluorescence intensity ratio <0.45). These data indicate that PPBs in these asymmetric divisions are not misoriented, but instead incompletely formed.

To confirm that TAN is a reliable proxy for prior PPB accumulation at the division site, we directly compared TAN1 accumulation with the microtubule PPB (Fig. 1C,E; Fig. S1). TAN1 localization mirrored the PPB type ∼97% of the time in wild-type siblings and ∼96% of the time in double mutants (*n*=81/84 wild-type cells; *n*=44/46 cells mutant cells; three plants for each genotype). Similar to previous research (Uyehara et al., 2024), there was a positive correlation between TAN1 and PPB mean fluorescence intensity in late G2/prophase (Fig. 1E; R^2^≥0.3 for three plants for each genotype). Furthermore, uneven TAN1 fluorescence intensities in p60 mutant cells during telophase occurred with similar frequency to uneven PPB accumulations during G2/prophase (37%, *n*=11/29 cells from three mutant plants versus 23% uneven PPBs, *P*=0.09 Chi-square test). Together, these data indicate that TAN1 is an accurate marker of the division site previously marked by the PPB.

**Fig. 2.**
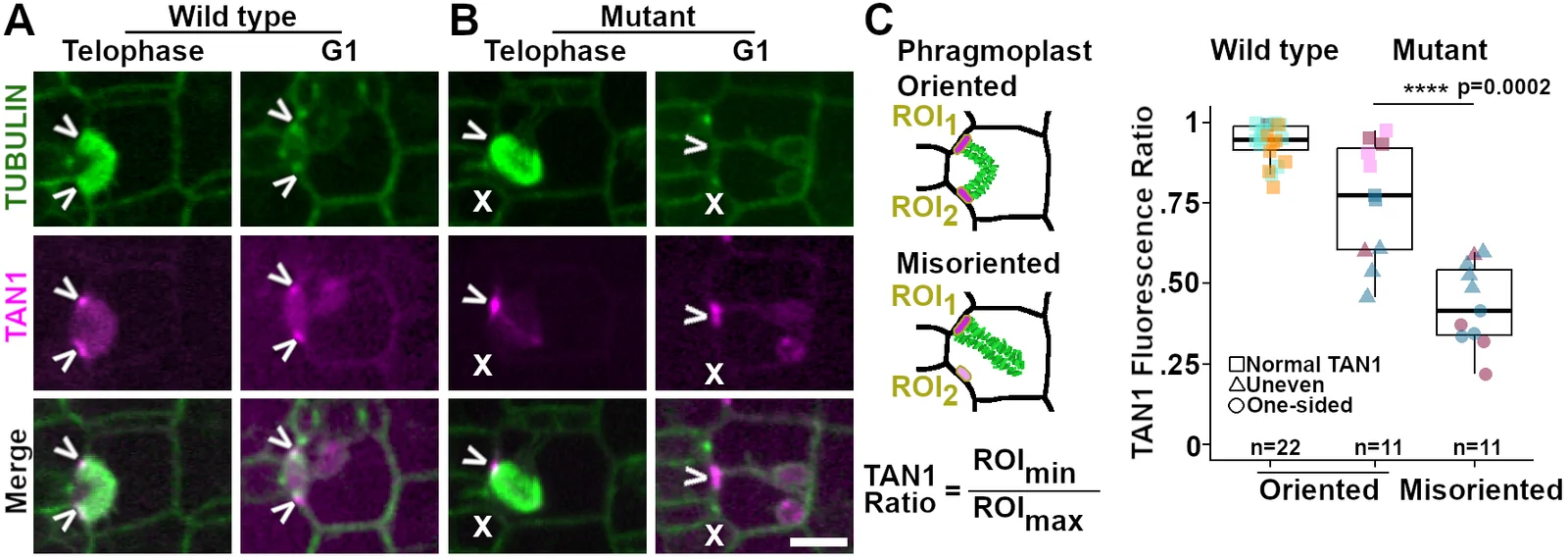
**Incomplete PPB accumulation leads to subsidiary mother cell asymmetric defects.** (A,B) Representative micrographs of asymmetrically dividing subsidiary mother cells in telophase or cytokinesis and early G1 expressing CFP–TUBULIN (microtubules) and TAN1–YFP (magenta). White arrowheads indicate the division site; the white Xs show division sites lacking TAN1. (A) Representative micrograph of wild-type cells with even TAN1 fluorescence at both sides of the division site and oriented phragmoplasts and (B) p60 mutants with one-sided TAN1 accumulation and a misoriented phragmoplast. All images are the same scale. Scale bar: 10 μm. (C) TAN1 fluorescence ratio for cells with oriented phragmoplasts: wild type (0.94±0.01), mutant (0.76±0.06); and misoriented phragmoplasts: mutant (0.43±0.04) (mean±s.e.m.). No misoriented phragmoplasts were observed in wild type. Three plants from each genotype were analyzed; each color denotes an individual plant. Cell counts are shown. *P*-value shown calculated from a Wilcoxon rank sum test. Boxplot shows median, first and third quartiles. Whiskers represent values within 1.5× the interquartile range from the first and third quartiles boundaries.

Next, we assessed TAN1 accumulation in telophase cells to determine whether incomplete PPBs caused misoriented subsidiary mother cell divisions. Misoriented phragmoplast ends were strongly correlated with local reduced TAN1 accumulation at the division site in p60 mutant cells. In wild-type siblings, phragmoplasts were correctly oriented and had a TAN1 fluorescence intensity ratio at the division site close to 1, indicating a prior normal PPB (Fig. 2A,C). In p60 mutants, we observed variable TAN1 fluorescence ratios during telophase indicative of normal, uneven and one-sided PPBs. Normal TAN1 fluorescence was correlated with correctly oriented phragmoplasts (Fig. 2C, TAN1 fluorescence ratio between 1 and 0.74, *n*=7/7). When TAN1 accumulation was only on one side of the division site, the phragmoplast was always oriented toward that side, and misoriented on the other side (Fig. 2B,C, TAN1 fluorescence ratio <0.45, *n*=6/6 one-sided TAN1 cells). In addition, in cells with uneven TAN1 accumulation, correct phragmoplast orientation was also correlated with the higher TAN1 accumulation (*n*=9/9), whereas the lower TAN1 accumulation division site side had both correctly oriented (*n*=4/9) and misoriented (*n*=5/9) phragmoplast ends. In other words, there were 11 misoriented phragmoplast ends that correlated with low or no TAN1 accumulation at the division site (Fig. 2C). This shows that one-sided PPBs, and occasionally uneven PPBs, cause misoriented subsidiary mother cell asymmetric divisions in the p60 mutant. Similarly, another mutant which forms incomplete (one-sided and uneven) PPBs, called *discordia1* (*dcd1*), has misoriented asymmetric subsidiary mother cell divisions that occur due to local absence of the PPB and lack of TAN1 accumulation (Uyehara et al., 2024). Taken together, this indicates that incomplete PPBs lead to asymmetric division positioning defects. More incomplete PPBs are observed during symmetric divisions than asymmetric divisions in the p60 mutant (41% versus 27%, respectively). However, incomplete PPBs do not cause misoriented symmetric divisions (Martinez et al., 2026). This indicates a different, and perhaps more crucial, role for the PPB in asymmetric division plane positioning.

**Fig. 3.**
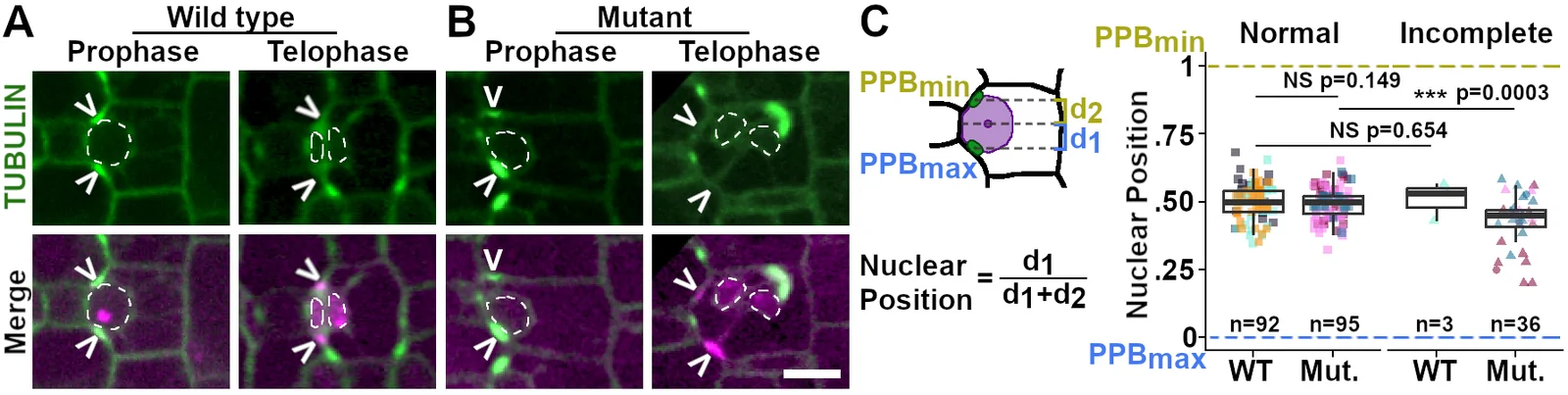
**Incomplete PPBs influence nuclear positioning towards microtubule accumulation.** (A,B) Representative micrographs of cells expressing CFP–TUBULIN (green) and TAN1–YFP (magenta) during prophase and telophase in (A) wild-type siblings and (B) p60 mutants. White arrowheads indicate the division site; nuclei are outlined with dotted lines. All images are the same scale. Scale bar: 10 μm. (C) Normalized nuclear position in cells with normal PPBs [wild type (WT), 0.50±0.01; mutant (Mut.), 0.49±0.01] and incomplete PPBs (wild type, 0.51±0.04; mutant, 0.43±0.02) (mean±s.e.m.). Three plants from each genotype were analyzed; each color denotes an individual plant. Shapes: square, normal PPB; triangle, uneven PPB; circle, one-sided PPB. *P*-value shown calculated from a Wilcoxon rank sum test. Boxplot shows median, first and third quartiles. NS, not significant. Whiskers represent values within 1.5× the interquartile range from the first and third quartiles boundaries.

Aberrant PPBs in symmetric divisions cause nuclear positioning defects in p60 mutants. To determine whether nuclear positioning is influenced by the PPB in subsidiary mother cell asymmetric divisions, the distance of the nucleus relative to the maximum PPB accumulation was measured (Fig. 3). Similar to what was seen in wild-type siblings, p60 mutant cells with normal PPBs had a mean nuclear position of ∼0.5, indicating that the nucleus was evenly centered between the two PPB accumulations (Fig. 3A,C). However, in double mutant cells with incomplete PPBs, the nucleus was closer to the maximum PPB accumulation (Fig. 3B,C, mean nucleus position ∼0.4). This indicates that nuclei in mutants were closer to the maximum PPB accumulation.

Nuclear polarization, or the movement of the nucleus towards the cell wall adjacent to the guard mother cell, occurs before PPB positioning in asymmetric divisions (Hazelwood et al., 2025). Nuclear polarization defects cause misoriented PPBs that later generate misoriented asymmetric divisions, as seen in the maize linc kash sine-like2 (*mlks2*) mutant (Ashraf et al., 2023; Gumber et al., 2019). *aladin1*, an additional maize mutant with subsidiary mother cell asymmetric division defects, encodes a component of the nuclear pore complex. Whether the defect is due to PPB or nuclear positioning, or something else, is currently unknown (Best et al., 2021)*.* Even though misoriented divisions were observed in p60 mutants, it was not due to a nuclear polarization defect because nuclei polarized correctly and PPBs were positioned at the expected division sites (Figs 2 and 3). This indicates that misoriented divisions in the p60 mutant are independent of nuclear polarization prior to PPB formation. It is currently unclear whether nuclear positioning defects after PPB formation contribute to division plane positioning but there is no evidence currently suggesting that it does.

**Fig. 4.**
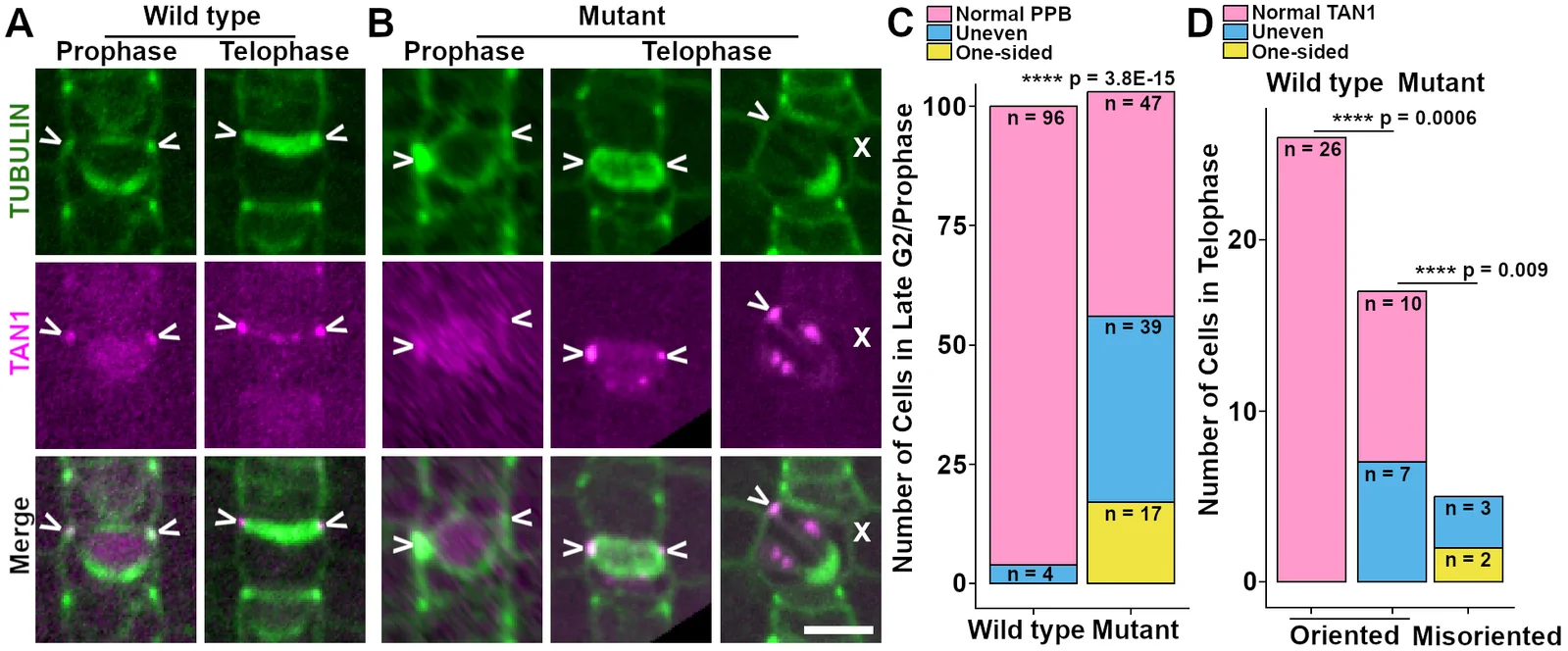
**Incomplete PPB accumulation sometimes leads to division positioning defects in guard mother cell precursors.** (A,B) Representative micrographs of asymmetrically dividing precursor cells expressing CFP–TUBULIN (green) and TAN1–YFP (magenta) showing (A) wild-type cells with even TAN1 fluorescence accumulation and oriented phragmoplast and (B) p60 mutant cells with uneven or one-sided TAN1 fluorescence with respective phragmoplast orientation. White arrowheads indicate the division site; white Xs show division sites lacking TAN1. All images are the same scale. Scale bar: 10 μm. (C) Stacked barplot of PPB accumulation observed in late G2/prophase cells. *P*-value shown calculated from a Chi-square test. (D) TAN1 accumulation and corresponding phragmoplast orientation. No misoriented phragmoplasts were observed in wild type. *P*-value shown calculated from a Fisher's exact test. Three wild-type and four p60 mutant plants were analyzed; n, cell counts.

To have a more general picture about the role of p60 proteins in asymmetric cell divisions, we examined another asymmetric division that generates the guard mother cell (Berg and Raissig, 2025; Facette and Smith, 2012; Guo et al., 2021). Unlike the highly curved subsidiary mother cell divisions, these asymmetric divisions occur transverse to the long axis of the cell, which is more similar to symmetric divisions. Compared to what was seen in wild type, there were significantly more misoriented guard mother cells in p60 mutants (∼8%, *n*=48/572 mutant cells versus 0.8%, *n*=6/551 wild-type cells; three plants per genotype, Welch's *t*-test *P*-value=7.8×10^−8^). To determine when the defect arises during this cell division, PPB and phragmoplast positioning was assessed. Normal PPBs were observed 96% of the time in wild-type cells (Fig. 4A,C, n=96/100 cells) and ∼46% of the time in p60 mutant cells (Fig. 4C, *n*=47/103 cells). No misoriented PPBs were observed in p60 mutant guard mother cell precursor cells. Instead, uneven (∼38%, Fig. 4B) and one-sided PPBs (∼16%) were observed. To determine whether these incomplete PPBs lead to defects in phragmoplast orientation, TAN1 accumulation was assessed in cells with oriented and misoriented phragmoplasts (Fig. 4D). Normal or even TAN1 accumulation at the division site was always correlated with oriented phragmoplasts in wild-type (*n*=26/26 cells) and p60 mutants (*n*=10/10 cells). In p60 mutant cells with uneven TAN1 division-site accumulation, 70% of phragmoplasts were correctly oriented (*n*=7/10 cells) whereas 30% were misoriented (*n*=3/10 cells). Rare p60 mutant cells with one-sided TAN1 accumulation had misoriented phragmoplasts (*n*=2/2 cells). This suggests that defective but not misoriented PPBs lead to misoriented divisions in the asymmetric division generating guard mother cells. Together, these results suggest that a fully formed PPB is more important for asymmetric divisions than symmetric divisions. This further implies that the geometry of the division might not be the primary cause of division positioning defects in asymmetric divisions.

## MATERIALS AND METHODS

### Plant growth conditions

Plants were grown under standard greenhouse conditions (32°C setpoint) with 4 h of supplemental lighting (∼400 μEm^−2^s^−1^) in 1 l pots with soil composed of 50% bark, 20% peat, 20% medium vermiculite and 10% perlite with calcium nitrate (75 ppm N and 90 ppm Ca), magnesium nitrate (50 ppm N and 45 ppm Mg), and Osmocote Classic 3-4M (NPK 14-14-14%, AICL SKU#E90550).

### Genotyping

DNA was extracted from leaves collected from 2-week-old plants using a standard protocol (Lunde, 2018; Mills et al., 2020). Mutant alleles (*dcd3a-2*, *dcd3b*) and transgenes (CFP-β-TUBULIN, TAN1-YFP; Mohanty et al., 2009) were genotyped using the primers and/or restriction enzymes specified previously (Martinez et al., 2026).

### Live-cell imaging of the stomatal asymmetric cell divisions

Plants expressing CFP–TUBULIN and TAN1–YFP were grown under standard greenhouse conditions for 4 weeks before dissecting to the youngest emerging leaf (ligule length <2 mm). Samples were cut 2–3 cm from the ligule and mounted with water into a rose chamber (Rasmussen, 2016). Images of the abaxial side of the leaf were obtained with a Hamamatsu 9100c EM-CCD camera using a Yokogawa W1 spinning disk microscope with Nikon Eclipse TE inverted stand. Solid-state Obis lasers (40–100 mW) of 445 nm or 514 nm with 480/40 or 540/30 nm emission filters (Chroma Technology) were used for CFP–TUBULIN and TAN1–YFP, respectively. A water immersion objective (60×/1.2 NA) and an ASI Piezo *Z*-stage was used to collect *Z*-stacks of 1–2 μm step size in Micromanager-1.4 using a three-axis DC controller. During image acquisition, unbiased imaging was performed by tiling across the leaf sample.

### PPB and TAN1 fluorescence intensity measurements

Using images taken as described above, the mean fluorescence intensity of the PPB (CFP–TUBULIN) and corresponding TAN1–YFP was obtained by outlining the PPB at the midplane of the cell using the polygon tool in FIJI software. The mean gray value of the same region of intertest (ROI) was measured in the corresponding CFP–TUBULIN and TAN1–YFP channels (Fig. 1C). For cells in telophase, TAN1–YFP at the division site was outlined at the midplane of the cell (Fig. 2C). For one-sided PPB or TAN1–YFP accumulations, a polygon was outlined at the other expected division site. PPB and TAN1–YFP accumulations in a cell were classified using the following fluorescence intensity percentage differences: normal <30%, uneven 30–75%, one-sided >75%. Fluorescence intensity ratios were obtained by dividing the minimum by the maximum fluorescence intensity and classified as normal >0.74, uneven 0.45–0.74, and one-sided <0.45 for both PPBs and TAN1. For Fig. 1, exposure times and gain conditions were different between different plant samples, therefore the slopes comparing TAN1–YFP and PPB fluorescence intensities are not comparable across samples.

### Nuclear positioning analysis

Using images of cells in late G2/prophase expressing CFP–TUBULIN, the nuclear and PPB centroids at the mid-plane of a cell were obtained in FIJI software. A straight line was drawn through the centroids and the distance between the lines was measured (Fig. 3C). For cells with one-sided PPBs, ROIs were traced at the expected location of the PPB. The normalized nuclear position was obtained by dividing the distance of the nucleus to the PPB with maximum fluorescence by the total distance between the PPBs (Fig. 3C).

### Figure preparation, graphs, and statistics

Gnu Image Manipulation Program (GIMP, v3.0.4, https://www.gimp.org/) was used for figure preparation. If applicable, levels were adjusted linearly and images were enlarged or rotated with no interpolation. Graphing and statistics were done in RStudio using the tidyr (https://tidyr.tidyverse.org/), ggplot2 (Wickham, 2016), paletteer (https://github.com/EmilHvitfeldt/paletteer), and ggprism (https://github.com/csdaw/ggprism) packages.

### Accessions

CFP–TUBULIN and TAN1–YFP lines were generated by the Maize Cell Genomics Group (Mohanty et al., 2009). Gene sequences can be found at MaizeGDB (https://www.maizegdb.org/gbrowse) using the following accession numbers: DISCORDIA 3A (Zm00001eb156560), DISCORDIA 3B (Zm00001eb360490). Maize lines are available on request.

## Supplementary Material



10.1242/joces.265055_sup1 Supplementary information
