# Supplementary material for: KATANIN is required for the formation of complete preprophase bands and subsequent division positioning in two asymmetric divisions of the maize stomatal complex
Source: J Cell Sci. 2026 Sep 10;139(17):jcs265055. doi: 10.1242/jcs.265055 (PMC13615675; doi:10.1242/jcs.265055)
Supplement: Supplementary file 1 [file joces-139-265055-s1.pdf]

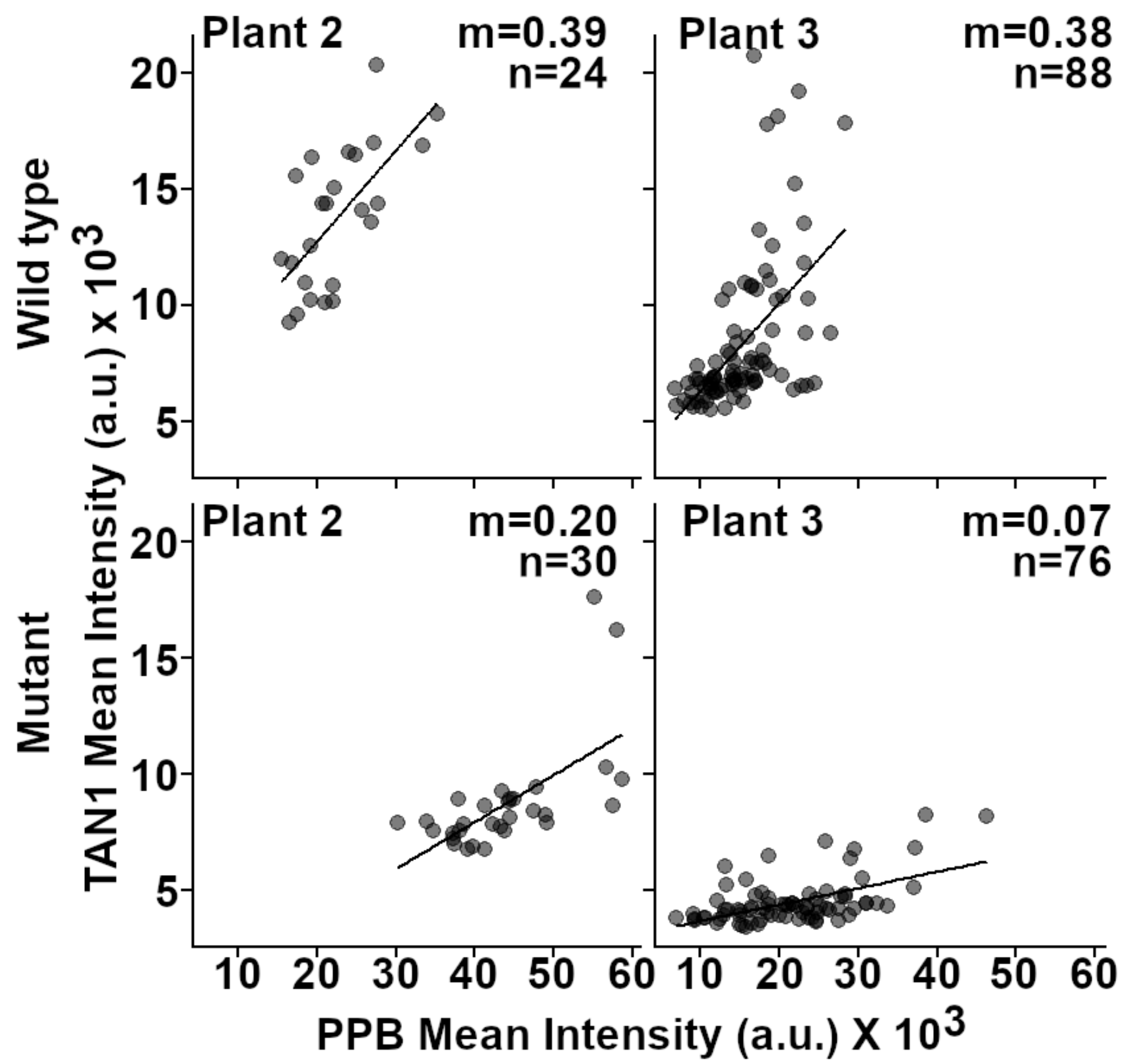

Fig. S1. TAN1 mean intensity accumulation correlates with PPB mean intensity. Raw data from two additional plants (see Figure 1C for Plant 1) with a black line of best fit (shown in Figure 1C as colored dotted lines); m = slope, n = number of division site fluorescence intensities measured.
