# Supplementary material for: KATANIN is required for the formation of complete preprophase bands and subsequent division positioning in two asymmetric divisions of the maize stomatal complex
Source: J Cell Sci. 2026 Sep 10;139(17):jcs265055. doi: 10.1242/jcs.265055 (PMC13615675; doi:10.1242/jcs.265055)
Supplement: Supplementary file 2 [file joces-139-265055_review_history.pdf]

KATANIN is required for the formation of complete preprophase bands and subsequent division positioning in two asymmetric divisions of the maize stomatal complex

Stephanie E. Martinez and Carolyn G. Rasmussen
DOI: 10.1242/jcs.265055

Editor: Charlotte Kirchelle

Review timeline
Original submission: 15 May 2026
Editorial decision: 24 June 2026
First revision received: 3 July 2026
Accepted: 28 July 2026

Original submission

First decision letter

MS ID#: jcs.265055

MS TITLE: KATANIN is required for the formation of complete preprophase bands and subsequent division positioning of subsidiary mother cells in maize

AUTHORS: Stephanie Martinez; Carolyn Rasmussen

ARTICLE TYPE: Short Report

Dear Dr Rasmussen,

We have now reached a decision on the above manuscript.

As you will see, the reviewers gave favourable reports but raised some critical points that will require amendments to your manuscript. I hope that you will be able to carry these out because I would like to be able to accept your paper, depending on further comments from reviewers.

Reviewer 1

The authors present a follow-up on their recent paper describing the role of KATANIN p60 on cell elongation and symmetric divisions in maize. Although the double mutant misspecified PPBs, it rarely affected cell division orientation in symmetric division. Here, the authors show that quite a large fraction of subsidiary mother cell (SMC) divisions are misoriented and that this is linked to incomplete (rather than misoriented) PPB formation, which then fails to guide phragmoplasts properly.

As it is usual with work from this lab, the cell biology is beautiful, appropriately executed, nicely illustrated, and the conclusions are well supported. I have only three rather minor concerns.

1 SMC divisions are not necessarily representative of asymmetric cell divisions (ACDs)
The authors present the ACD of SMCs as representative of ACDs in general. I would, however, argue that the SMC division is particularly spectacular, with extreme asymmetry and peculiar PPB positioning. If the authors want to make a general statement about ACDs vs. SCDs (e.g. page 5, line

© 2026. Published by The Company of Biologists under the terms of the Creative Commons Attribution License (https://creativecommons.org/licenses/by/4.0/).

55), then earlier protodermal cells that undergo a more "representative" transverse ACD should be analyzed as well. I think that the extreme asymmetric division of SMCs is a sensitized system that can teach us many aspects of asymmetry and polarity, but I do not think of it as representative of ACDs. No additional experiments are necessary, but this aspect should be acknowledged.

2 Figure 1
The ratio differences between upper and lower PPB were only explained in the text (page 5, first paragraph), but not actually visualized in a plot. To see the distribution of the ratio, an actual jitter and boxplot might be a nice addition to visualize the PPB formation defects.
In addition, I don't understand why Figure 1C only represents one individual for the jitter data and does not show all three individuals together and a single trendline per genotype. Could the authors elaborate on their reasoning?
Also, the difference in slope between mutant and WT (shallower in mutant) might actually have a biological meaning, where PPB formation and cortical division site maintenance might be off in the p60 mutants.

3 No ectopic cortical division sites formed in p60 SMCs?
The authors showed previously that ectopic cortical division sites were formed in p60 mutants (3-sided PPBs in Martinez et al. 2026), but say that this is not the case in SMCs. The only mutant shown to form ectopic cortical division sites in grass SMCs is the polar mutant in Brachypodium distachyon. I wonder how the authors interpret the absence of ectopic sites in p60 SMCs compared to other cells and how they interpret this observation in respect to the ectopic cortical division sites formed in polar mutants.

Reviewer 2

SUMMARY OF THE ADVANCE MADE IN THIS PAPER AND ITS POTENTIAL SIGNIFICANCE TO THE FIELD

This short report summarizes subcellular phenotypes linked to the loss of the microtubule severing protein katanin in maize. It was found that subsidiary mother cells often formed informatively oriented but partially defective preprophase band (PPB) microtubule array. The wild-type cells produce PPB with uniform microtubule bundles as represented by the so-called two accumulations in the middle focal plane but the katanin mutant cells often have one instead of two such accumulations. It was concluded that normal PPB accumulations were required and competent for attracting the expanding phragmoplast edge and prophase nucleus during asymmetric divisions.

The story is straightforward.

SUGGESTIONS TO AUTHORS

Major comments [Please request additional experiments only if they are essential for supporting the conclusions; authors should be encouraged to highlight any claims that are preliminary or speculative, or to discuss any pitfalls or alternative interpretations in a 'Limitations' section]

Minor comments
The authors described the targeted division as subsidiary cell division in several places. Changes to subsidiary mother cell divisions are probably needed.

Their recent paper reports that similar defective PPBs brought about by the katanin mutation do not seem to compromise phragmoplast guidance in pavement cells undergoing symmetric division. The difference seemed to imply that defective PPB was still competent for guiding phragmoplast expansion during symmetric divisions. One may ask what the differences in the PPB-dependent guidance mechanism would be under two conditions. Would the asymmetric division of subsidiary mother cells be more challenging than symmetric divisions?

The n values in Figure 2C do not match those in the text unless there are misunderstandings here.

Figure 3 summarizes differences in nuclear centering in mutant and wild-type cells. The phenotype is interesting. But one may ask how the decentered nuclei may be related to defective divisions in these cells.

© 2026. Published by The Company of Biologists under the terms of the Creative Commons Attribution License (https://creativecommons.org/licenses/by/4.0/).

First revision

Author response to reviewers' comments

Here we would like to gratefully acknowledge the reviewers for their helpful comments, as they have, in our opinion, substantially strengthened the paper. Responses to reviewer comments are in blue. When new text was added to the manuscript, we appended it here in red.

Comments from the Reviewers:

Reviewer 1: The authors present a follow-up on their recent paper describing the role of KATANIN p60 on cell elongation and symmetric divisions in maize. Although the double mutant misspecified PPBs, it rarely affected cell division orientation in symmetric division. Here, the authors show that quite a large fraction of subsidiary mother cell (SMC) divisions are misoriented and that this is linked to incomplete (rather than misoriented) PPB formation, which then fails to guide phragmoplasts properly.

As it is usual with work from this lab, the cell biology is beautiful, appropriately executed, nicely illustrated, and the conclusions are well supported. I have only three rather minor concerns.

1 SMC divisions are not necessarily representative of asymmetric cell divisions (ACDs)
The authors present the ACD of SMCs as representative of ACDs in general. I would, however, argue that the SMC division is particularly spectacular, with extreme asymmetry and peculiar PPB positioning. If the authors want to make a general statement about ACDs vs. SCDs (e.g. page 5, line 55), then earlier protodermal cells that undergo a more "representative" transverse ACD should be analyzed as well. I think that the extreme asymmetric division of SMCs is a sensitized system that can teach us many aspects of asymmetry and polarity, but I do not think of it as representative of ACDs. No additional experiments are necessary, but this aspect should be acknowledged.

Thank you for raising this excellent point, which we answer below.

We analyzed the role of KATANIN in the transverse asymmetric cell division that generates guard mother cells: these cells made incomplete PPBs but did not make ectopic or misoriented PPBs. Misoriented divisions were strongly correlated with lack of TAN1 at the division site during telophase, suggesting that earlier failure to establish a PPB was the proximal cause. Below we append the added text and new figure.

“To have a more general picture about the role of p60 in asymmetric cell divisions, we examined another asymmetric division that generates the guard mother cell (Guo et al. 2021; Berg and Raissig 2025; Facette and Smith 2012). Unlike the highly curved subsidiary mother cell divisions, these asymmetric divisions occur transverse to the long axis of the cell, more similar to symmetric divisions. Compared to wild type, there were significantly more misoriented guard mother cells in *p60* mutants (~ 8%, n = 48/572 mutant cells vs. 0.8%, n = 6/551 wild-type cells; 3 plants per genotype, Welch’s t-test p-value = 7.8E-8). To determine when the defect arises during this cell division, PPB and phragmoplast positioning was assessed. Normal PPBs were observed 96% of the time in wild-type cells (Figure 4A, C, n = 96/100 cells) and ~46% of the time in *p60* mutant cells (Figure 4C, n = 47/103 cells). No misoriented PPBs were observed in *p60* guard mother cell precursor cells. Instead, uneven (~38%, Figure 4B) and one-sided PPBs (~16%) were observed. To determine if these incomplete PPBs lead to defects in phragmoplast orientation, TAN1-YFP at the division site, as a proxy for former PPB accumulation, was assessed in cells with oriented and misoriented phragmoplasts (Figure 4D). Normal or even TAN1 accumulation at the division site was always correlated with oriented phragmoplasts in wild type (n = 26/26 cells) and *p60* mutants (n = 10/10 cells). In *p60* cells with uneven TAN1 division-site accumulation, 70% of phragmoplasts were correctly oriented (n = 7/10 cells) while 30% were misoriented (n = 3/10 cells). Rare *p60* cells with one-sided TAN1 accumulation had misoriented phragmoplasts (n = 2/2 cells). This suggests that defective but not misoriented PPBs lead to misoriented divisions in the asymmetric division

© 2026. Published by The Company of Biologists under the terms of the Creative Commons Attribution License (https://creativecommons.org/licenses/by/4.0/).

generating guard mother cells. Together, these results suggest that fully formed PPBs are more important for asymmetric division positioning compared to symmetric division positioning. It further implies that the geometry of the division may not be the primary cause of division positioning defects in asymmetric divisions.

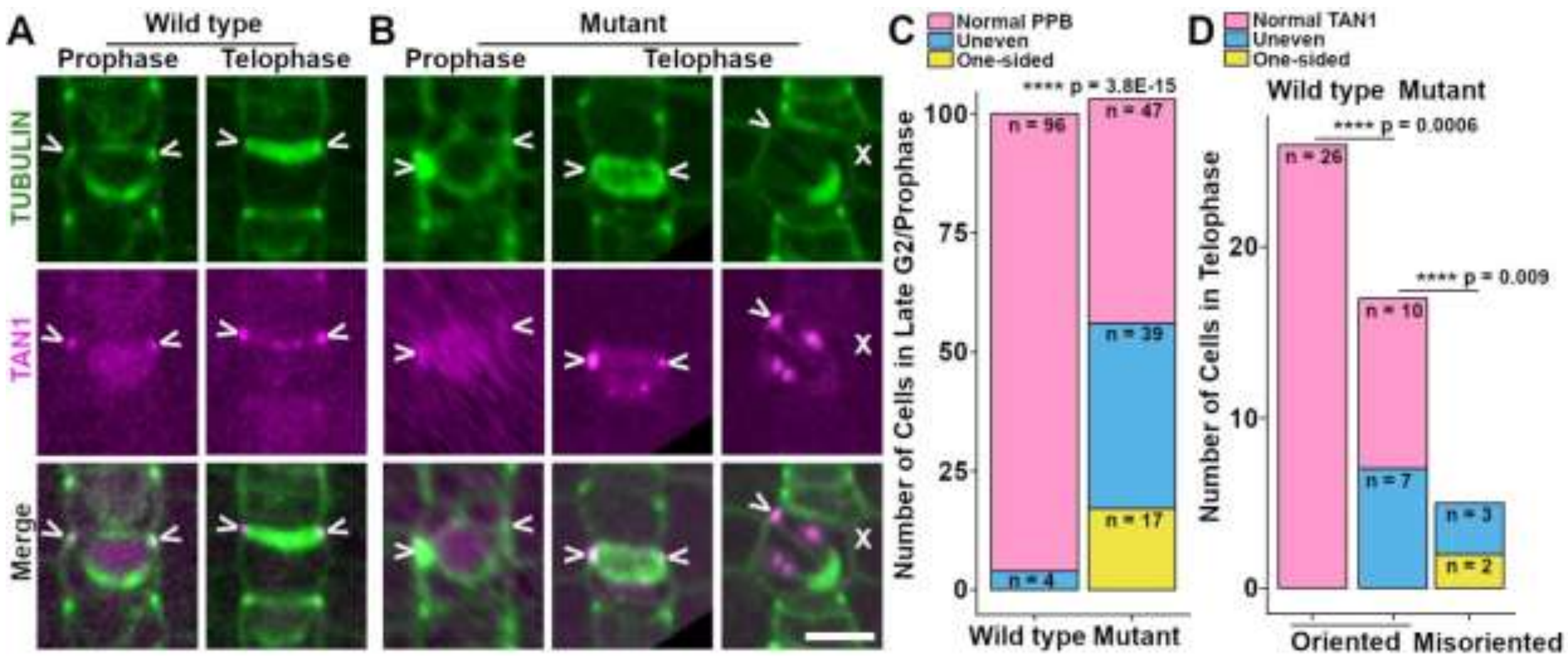

Figure 4. Incomplete PPB accumulation sometimes leads to division positioning defects in guard mother cell precursors. A, B) Representative micrographs of asymmetrically-dividing precursor cells expressing CFP-TUBULIN (green) and TAN1-YFP (magenta) showing A) wild-type cells with even TAN1 fluorescence accumulation and oriented phragmoplast and B) *p60* mutant cells with uneven or one-sided TAN1 fluorescence with respective phragmoplast orientation. White arrowheads indicate the division site, white Xs show division sites lacking TAN1. All images are the same size; scale bar = 10 µm. C) Stacked barplot of PPB accumulation observed in late G2/prophase cells. Chi-square test p-value shown. D) TAN1 accumulation and corresponding phragmoplast orientation. No misoriented phragmoplasts were observed in wild type. Fisher's exact test p-values shown. 3 wild-type and 4 *p60* mutant plants were analyzed, n = cell counts.

2 Figure 1: The ratio differences between upper and lower PPB were only explained in the text (page 5, first paragraph), but not actually visualized in a plot. To see the distribution of the ratio, an actual jitter and boxplot might be a nice addition to visualize the PPB formation defects.

Thank you for this helpful suggestion. We have now added PPB fluorescence intensity ratios to Figure 1 with the following changes to the text and figure legend (both are appended below for your reference). Additionally, we have altered the text to use the ratio rather than the % difference, with the explicit purpose of comparing these results to those results presented in Figure 2.

"We defined normal PPBs as those with uniform fluorescence intensities on both sides of the division site at the midplane of the cell ~~with<30%difference~~ with PPB intensity ratios from 1 to 0.74 (Figure 1A, C, D). Normal PPBs were observed 97% of the time in wild-type plants (n = 95/98 cells from 3 plants) and 73% of the time in *p60* mutants (n = 102/139 cells from 3 plants). The rest of the PPBs in the *p60* mutant were either uneven (~23%, n = 32/139 cells ~~with 30-75%~~ fluorescence intensity ~~percent difference~~ ratio between 0.45-0.74 at the division site, Figure 1B) or one-sided (~4%, n =5/139 cells with fluorescence intensity ~~differences > 75% and~~ ratio < 0.45). These data indicated that PPBs in these asymmetric divisions were not misoriented, but instead incompletely formed."

© 2026. Published by The Company of Biologists under the terms of the Creative Commons Attribution License (https://creativecommons.org/licenses/by/4.0/).

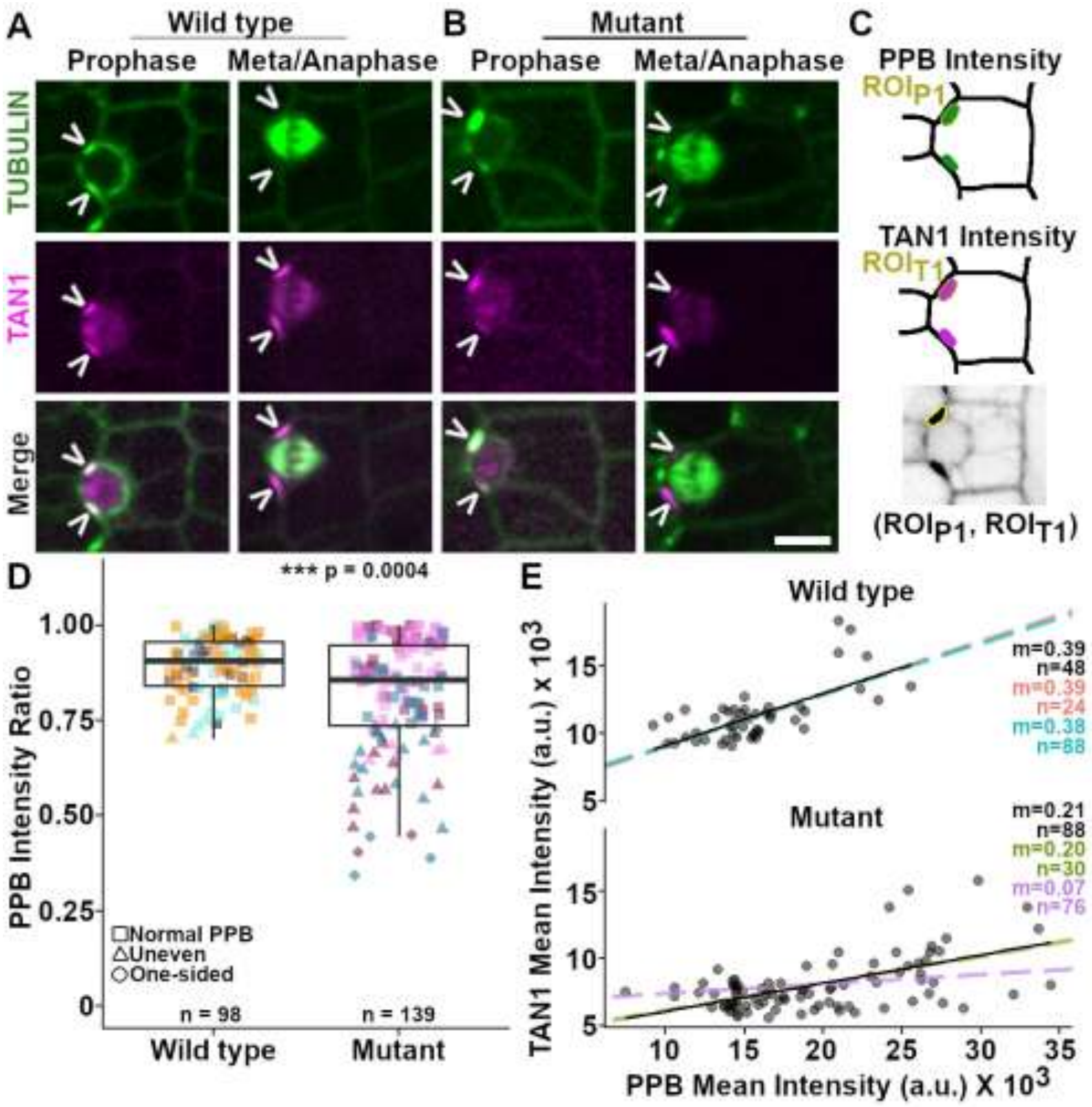

Figure 1. TAN1 is a proxy for PPB accumulation. A, B) Representative micrographs of asymmetrically-dividing subsidiary mother cells expressing CFP-TUBULIN (microtubules, green) and TAN1-YFP (magenta) for A) wild-type cells (*dcd3a-2/+; dcd3b-1/+*) with equivalent accumulation of both microtubules and TAN1-YFP and B) *p60* mutant cells (*dcd3a-2; dcd3b-1)* with uneven accumulation of both TAN1-YFP and microtubules at each side of the division site. White arrowheads indicate the division site. All images are the same size; scale bar = 10 µm. C) Schematic showing the division site where fluorescent intensities were measured. D) PPB fluorescence intensity ratio from three wild-type and mutant plants, indicated with different color dots. Dot shape = PPB type. Wilcoxon rank sum test p-value and cell counts shown. Boxplot shows median, first and third quartiles, and whiskers ≤ 1.5X the interquartile range. E) Scatter plots comparing relative TAN1 and PPB mean intensity from cells in late G2/prophase. Three plants per genotype were analyzed. Raw data from one plant consists of black dots corresponding to individual fluorescence intensity measurements (with a black line of best fit); colored dotted lines = best fit of 2 other plants. m = slope, n = number of division site fluorescence intensities measured."

In addition, I don't understand why Figure 1C only represents one individual for the jitter data and does not show all three individuals together and a single trendline per genotype. Could the authors elaborate on their reasoning? Also, the difference in slope between mutant and WT (shallower in mutant) might actually have a biological meaning, where PPB formation and cortical division site maintenance might be off in the p60 mutants.

The reason all three individuals were not plotted is due to differences in image acquisition conditions (different exposure and gain) between plant samples. The difference in exposure times between channels would also alter the apparent slope. Due to this, we are unable to conclude whether or not TAN1 is recruited differently in the *p60* mutants. What we can (and do) conclude is that TAN1 fluorescence intensities are correlated with PPB fluorescence intensities. Therefore, we

© 2026. Published by The Company of Biologists under the terms of the Creative Commons Attribution License (https://creativecommons.org/licenses/by/4.0/).

chose to show one individual for the jitter data and normalized the trendlines for each individual measured. To show the data of the other individuals, we now add Supplementary Figure 1 to the manuscript (appended below) and additional explanatory text in the methods:

"For Figure 1, exposure times and gain conditions were different between different plant samples, therefore the slopes comparing TAN1-YFP and PPB fluorescence intensities are not comparable across samples."

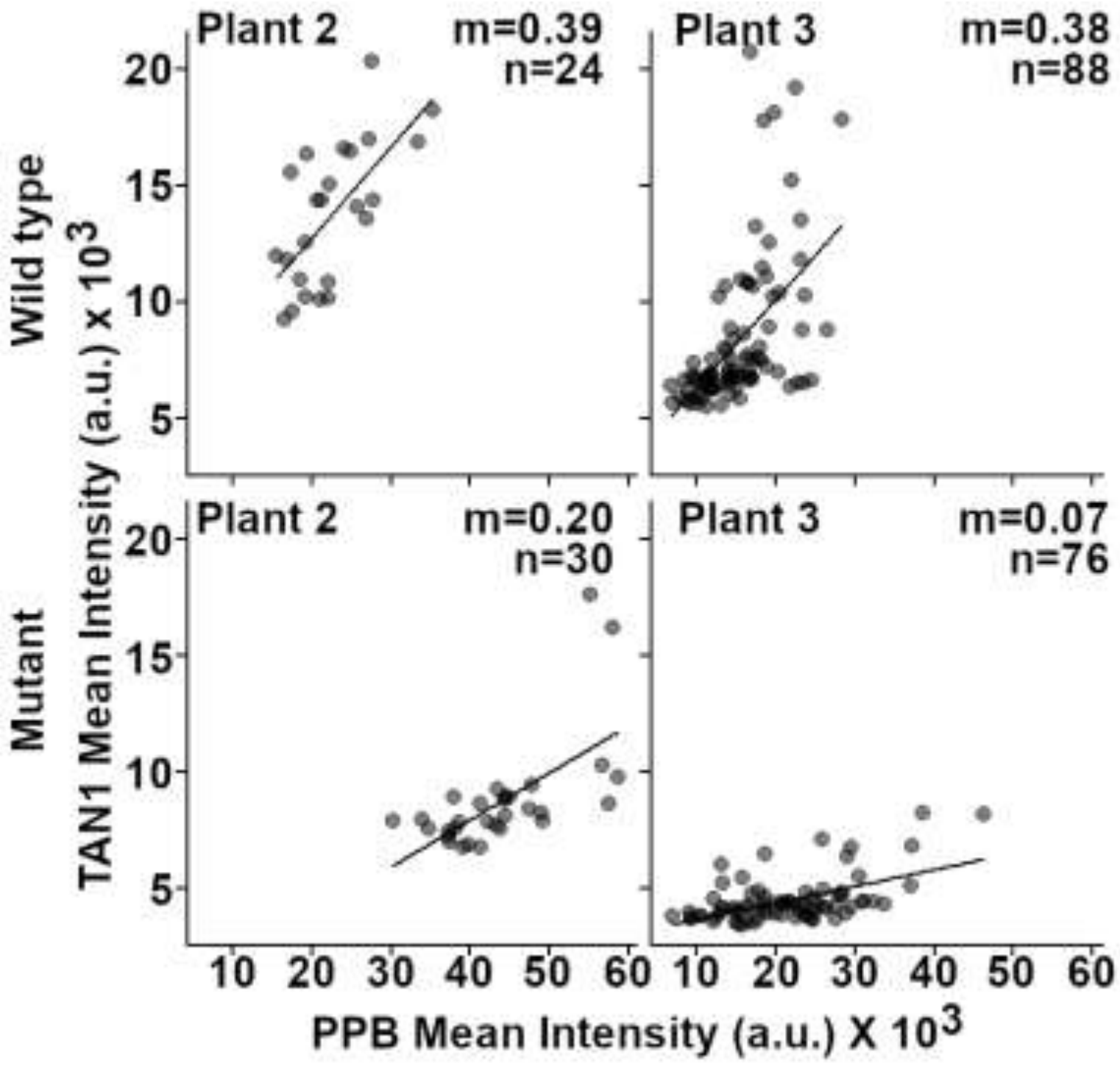

Supplementary Figure 1. TAN1 mean intensity accumulation correlates with PPB mean intensity. Comparative intensity measurements from two additional plants (see Figure 1C for Plant 1) with a black line of best fit (shown in Figure 1C as colored dotted lines); m = slope, n = number of division site fluorescence intensities measured.

3 No ectopic cortical division sites formed in p60 SMCs?
The authors showed previously that ectopic cortical division sites were formed in p60 mutants (3-sided PPBs in Martinez et al. 2026), but say that this is not the case in SMCs. The only mutant shown to form ectopic cortical division sites in grass SMCs is the polar mutant in Brachypodium distachyon. I wonder how the authors interpret the absence of ectopic sites in p60 SMCs compared to other cells and how they interpret this observation in respect to the ectopic cortical division sites formed in polar mutants.

We have now added a sentence to the introduction describing two mutants representing two types of aberrant division positioning defects in SMCs, including *Bdpolar:* "Misoriented subsidiary mother cell PPBs have been observed in maize *brick1* mutants (Panteris et al. 2006), while *B. distachyon Bdpolar* mutant subsidiary mother cells sometimes have additional division sites specified by *BdTAN1* (Zhang et al. 2022)."

There were occasional 3-sided PPBs in symmetric pavement cell divisions, but they were not observed more frequently in *p60* mutants than in wild-type cells (Martinez et al 2026). We concluded that 3-sided PPBs were not due to the *p60* mutant. Instead, we observed rare misoriented, curved PPBs that generate an aberrant curved division. No misoriented or ectopic PPBs were observed in either the GMC precursor division or the SMC division.

Reviewer 2: SUMMARY OF THE ADVANCE MADE IN THIS PAPER AND ITS POTENTIAL SIGNIFICANCE TO THE FIELD

© 2026. Published by The Company of Biologists under the terms of the Creative Commons Attribution License (https://creativecommons.org/licenses/by/4.0/).

This short report summarizes subcellular phenotypes linked to the loss of the microtubule severing protein katanin in maize. It was found that subsidiary mother cells often formed informatively oriented but partially defective preprophase band (PPB) microtubule array. The wild-type cells produce PPB with uniform microtubule bundles as represented by the so-called two accumulations in the middle focal plane but the katanin mutant cells often have one instead of two such accumulations. It was concluded that normal PPB accumulations were required and competent for attracting the expanding phragmoplast edge and prophase nucleus during asymmetric divisions.

The story is straightforward.

SUGGESTIONS TO AUTHORS

Major comments [Please request additional experiments only if they are essential for supporting the conclusions; authors should be encouraged to highlight any claims that are preliminary or speculative, or to discuss any pitfalls or alternative interpretations in a 'Limitations' section]

Minor comments
The authors described the targeted division as subsidiary cell division in several places. Changes to subsidiary mother cell divisions are probably needed.
Thank you for pointing this out. We have changed “subsidiary cell division” to “subsidiary mother cell division” throughout the text and the figure 2 legend.

Their recent paper reports that similar defective PPBs brought about by the katanin mutation do not seem to compromise phragmoplast guidance in pavement cells undergoing symmetric division. The difference seemed to imply that defective PPB was still competent for guiding phragmoplast expansion during symmetric divisions. One may ask what the differences in the PPB-dependent guidance mechanism would be under two conditions. Would the asymmetric division of subsidiary mother cells be more challenging than symmetric divisions?
This is an insightful question, and one that we have been curious about for a long time. Your question, along with Reviewer 1’s question, prompted us to assess another asymmetric division that occurs during maize stomatal complex formation, the division that generates the guard mother cell. Our prior assumption was that the **geometry** of the SMC division, with a highly curved PPB and phragmoplast, was what increased reliance on the PPB. However, we now know that this other asymmetric division, which does not generate a curved cell, also relies on an intact PPB for division positioning. Please see our response to Reviewer 1’s first question above.

The n values in Figure 2C do not match those in the text unless there are misunderstandings here.

Thank you for the helpful comment. To reduce misunderstandings, the following changes were made to the text:

“Next, we assessed TAN1 accumulation in telophase cells to determine whether incomplete PPBs caused misoriented asymmetric divisions. Misoriented phragmoplast ends were strongly correlated with local reduced TAN1 accumulation at the division site in the *p60* mutant. In wild-type siblings, phragmoplasts were correctly oriented and had a TAN1 fluorescence intensity ratio at the division site close to 1, indicating a prior normal PPB (Figure 2A, C). In *p60* mutants, we observed variable TAN1 fluorescence ratios during telophase indicative of normal, uneven, and one-sided PPBs. Normal TAN1 fluorescence was correlated with correctly oriented phragmoplasts (Figure 2C, TAN1 fluorescence ratio > 0.74, n = 7/7). When TAN1 accumulation was only on one side of the division site, the phragmoplast was always oriented toward that side, and misoriented on the other side (Figure 2B, C, TAN1 fluorescence ratio < 0.45, n = 6/6 one-sided TAN1 cells). In addition, in cells with ~~whentherewas~~ uneven TAN1 accumulation, correct phragmoplast orientation was also correlated with the higher TAN1 accumulation (n = 9/9), while the lower TAN1 accumulation division site side had both correctly oriented (4/9) and misoriented (5/9) phragmoplast ends. In other words, there were 11 misoriented phragmoplast ends correlated with low or no TAN1 accumulation at the division site ~~Sometimes,unevenTAN1 accumulationdid notleadtoamisorientedphragmoplast(n=4/9cells)~~ (Figure 2C). This shows that one-sided PPBs, and occasionally uneven PPBs, cause misoriented subsidiary mother cell asymmetric divisions in the

© 2026. Published by The Company of Biologists under the terms of the Creative Commons Attribution License (https://creativecommons.org/licenses/by/4.0/).

p60 mutant."

Figure 3 summarizes differences in nuclear centering in mutant and wild-type cells. The phenotype is interesting. But one may ask how the decentered nuclei may be related to defective divisions in these cells.

We were unable to determine whether nuclei centering influences division plane positioning from our analysis. The following sentence addresses this point in the text:

"It is currently unclear whether nuclear positioning defects after PPB formation contribute to division plane positioning but there is no evidence currently suggesting that it does."

Second decision letter

MS ID#: jcs.265055R1

MS Title: KATANIN is required for the formation of complete preprophase bands and subsequent division positioning in two asymmetric divisions of the maize stomatal complex

Authors: Stephanie Martinez; Carolyn Rasmussen

Article Type: Short Report

Dear Dr Rasmussen,

Thank you for revised manuscript. I am happy to tell you that your manuscript has been accepted for publication in Journal of Cell Science, pending standard publication integrity checks.

Reviewer 1

I would like to thank the authors for the additional, beautiful data that was included and for the detailed explanations that fully addressed all my concerns. I have no further comments.

Reviewer 2

SUMMARY OF THE ADVANCE MADE IN THIS PAPER AND ITS POTENTIAL SIGNIFICANCE TO THE FIELD

The authors reported a critical role of katanin in organization of the PPB microtubule array.

SUGGESTIONS TO AUTHORS

The authors have adequately addressed comments made by this reviewer.

Major comments [Please request additional experiments only if they are essential for supporting the conclusions; authors should be encouraged to highlight any claims that are preliminary or speculative, or to discuss any pitfalls or alternative interpretations in a 'Limitations' section]

Minor comments

© 2026. Published by The Company of Biologists under the terms of the Creative Commons Attribution License (https://creativecommons.org/licenses/by/4.0/).
